# The impact of chronic illness resources, fear of recurrence, hope, and health locus of control on self-management behaviors in post-stroke patients: a cross-sectional study

**DOI:** 10.3389/fmed.2025.1598945

**Published:** 2025-05-20

**Authors:** Jingxia Cheng, Xiaolian Jiang, Xi Liao, Lili Zhou, Li Qin, Hongying Liu

**Affiliations:** ^1^Division of Pancreatic Surgery, Department of General Surgery, West China Hospital, Sichuan University/West China School of Nursing, Sichuan University, Chengdu, China; ^2^West China School of Nursing, Sichuan University/West China Hospital, Sichuan University, Chengdu, China; ^3^Colorectal Cancer Center, Department of General Surgery, West China Hospital, Sichuan University/West China School of Nursing, Sichuan University, Chengdu, China

**Keywords:** stroke, self-management (self-care), chronic illness resources, fear of recurrence, hope, health locus of control (HLC)

## Abstract

**Background:**

Stroke often leads to functional impairment in patients and carries a risk of recurrence. Therefore, it is crucial for post-stroke patients to proactively pursue self-management behaviors that foster functional recovery and prevent recurrent strokes, such as keeping a balanced diet or exercising. The implementation of self-management behaviors requires the combined influence of internal and external factors, which have not been fully explored. This study aimed to investigate the level of self-management behaviors in post-stroke patients and to explore the impact of chronic illness resources, fear of recurrence, hope, and health locus of control on these behaviors.

**Methods:**

A cross-sectional study was conducted among 262 post-stroke patients from a tertiary hospital in Sichuan, China. The scores of the Self-management Behavior Scale for Post-stroke patients, the Chronic Illness Resource Scale, the Fear of Recurrence Inventory Short Form, the Herth Hope Index, and the Multidimensional Health Locus of Control were collected. Multiple linear regression was ultimately used to determine the predictors of self-management behaviors.

**Results:**

The mean score of the Self-management Behavior Scale for Post-stroke patients was 92.23 ± 14.61, with the item mean score of 3.55 ± 0.56. Approximately 56.2% of the variation in self-management behaviors in post-stroke patients could be explained by educational level (β = 0.222, *P* < 0.001), rehabilitation training (β = 0.444, *P* < 0.001), physician/health care team resources (β = 0.139, *P* = 0.007), family and friends resources (β = 0.160, *P* = 0.003), personal resources (β = 0.182, *P* = 0.002), fear of recurrence (β = –0.145, *P* = 0.001), internal locus of control (β = 0.181, *P* < 0.001), and chance health locus of control (β = –0.141, *P* = 0.004).

**Conclusion:**

The self-management of post-stroke patients was moderate, with emotional management and utilization of resources being the weaknesses. Patients’ coping abilities, the establishment of a positive psychological locus of control, and the development of a realistic yet constructive recurrence risk perception were beneficial for their self-management. In addition, professional rehabilitation treatment and enhanced support from chronic illness resources such as medical staff and family and friends were also important.

## 1 Introduction

Stroke is the second leading cause of death and the third leading cause of disability-adjusted life years (DALYs) among non-communicable diseases globally, with over 80% of stroke-related DALYs occurring in low-income and middle-income countries (1). In China, the prevalence of stroke ranks first globally and is continuously increasing, primarily driven by an aging population and lifestyle changes (2).

The heavy disease burden associated with stroke is exacerbated by its high disability rate, which ranges from 11.1 to 29.2% within the first 12 months (3). A considerable proportion of post-stroke patients experience varying degrees of cognitive, linguistic, motor, swallowing, excretory, and other functional impairments. Equally concerning is the recurrence of stroke, a primary contributor to long-term disability among patients and an increase in mortality rates (4). The recurrence rate of stroke was reported as 5.7% (5.5–6.0%) for stroke survivors, ranging from 2.5% for subarachnoid hemorrhage to 6.4% for ischemic stroke (5), and the recurrence rate of patients with cerebral infarction can be as high as 11.65% (2).

Thus, for post-stroke patients, functional recovery and the prevention of recurrence emerge as primary health objectives. A systematic review has shown that smoking, HT, DM, AF, prior cerebrovascular event, and increased severity of stroke were independent risk factors of recurrent stroke (6). The performance of functional exercises, along with the prevention of recurrence risk factors like smoking cessation, demands that patients foster healthy behaviors via self-regulation and resource utilization.

Research on self-management began in the 1960s and has since evolved to encompass various definitions and connotations (7–10). Generally, it involves patients managing their disease cognition, behavior, roles, and emotions; mastering problem-solving skills related to health issues; setting health goals; making informed health decisions; utilizing available health resources; and collaborating with healthcare professionals (11, 12). Self-management has gradually been integrated into the management of chronic diseases such as hypertension, diabetes, maintenance hemodialysis, and stroke. Both domestic and international studies have demonstrated that self-management positively influences outcomes for stroke patients (13, 14). However, in China, the level of self-management among post-stroke patients remains suboptimal (15–17).

Previous research has identified several factors influencing self-management in post-stroke patients (18–20). Internal patient factors primarily include knowledge about stroke, self-efficacy, disease perception, coping abilities, perceived burden of illness, and emotional states. Conversely, investigations into external factors have largely focused on a singular dimension—such as family support or social support—leaving many areas unexplored regarding the determinants of self-management behaviors in post-stroke patients.

Considering the profound influence of the interplay between individuals and their surroundings on health behaviors, it is crucial to take into account the influencing factors of self-management behaviors, encompassing both personal characteristics and external resources. In this vein, Glasgow’s Social Ecological Model (SEM) offers a comprehensive framework, categorizing the social resources that affect the self-management of chronic disease patients into four distinct levels and seven aspects (21). Personal factors encompass a range of internal elements, including genetic predisposition, attitudes, beliefs, personality traits, coping mechanisms, and self-efficacy. Intimate relationships involve the support provided by health service teams, such as nursing and medical professionals, as well as family members or friends. The environmental level considers various settings, such as the workplace, organizations, neighborhoods, and the physical surroundings. Cultural aspects, on the other hand, encompass media, policies, and broader community or regional resources. The Chronic Illness Resources Scale (CIRS), which was developed based on the Social Ecological Model, has gained widespread acceptance as a tool to assess the health resource status of chronic disease patients (22–24). However, there is a notable lack of research on this topic specifically concerning stroke survivors.

Although external factors are undoubtedly important, the inner strength and resilience of patients remain the ultimate determinants of their self-management behaviors. In light of this, there is a pressing need for further exploration of the impact of additional critical variables on patients’ self-management behaviors.

As a consequence of the high rates of recurrence and disability associated with stroke, fear of recurrence has emerged as a significant stressor for patients (25). This phenomenon is often characterized by the degree of apprehension and concern regarding disease recurrence and progression (26). The intensity of fear related to recurrence can vary from a typical emotional response to the illness to an overwhelming sense of dread (27). At lower levels, this fear might be regarded as a normal and transient emotional reaction that fosters appropriate vigilance concerning potential disease recurrence while promoting effective health management strategies. However, when patients’ fears surpass the actual risk of recurrence, they might develop intrusive thoughts that exacerbate their emotional burden (28). Therefore, exploring how fear of recurrence shapes patient self-management behaviors is crucial. Unfortunately, few studies have specifically addressed this issue in post-stroke patients.

Hope, as an intrinsic strength, exerts a positive influence on patients’ emotional regulation and health-related behaviors. Relevant research has focused on populations such as those with cancer, chronic pain, critical illnesses, terminal conditions, and the elderly (29). Studies have demonstrated that hope in post-stroke patients is positively correlated with their psychological resilience (30), coping abilities (31), and self-efficacy (32). However, only a limited number of researchers have incorporated this aspect into studies examining self-management among stroke patients.

The health locus of control theory proposed by Rotter posits that the locus of control is a significant personality variable influencing patients’ engagement in health management behaviors (33). Its impact on self-management behaviors has been substantiated in chronic disease populations, including those with hemodialysis (34), diabetes (35), and coronary heart disease (36). However, there is a paucity of relevant research focusing on post-stroke patients. Individuals with an internal locus of control (ILC) are more likely to believe that their health outcomes are contingent upon their own actions and, thus, tend to adopt healthier behaviors. Conversely, individuals who exhibit a powerful others’ health locus of control (PHLC) often perceive their health as being influenced by healthcare professionals—such as medical staff or family members—and are, therefore, more inclined to adhere to external advice. Those with a chance health locus of control (CHLC), however, attribute their health outcomes to luck and consequently might be less motivated to initiate behavioral changes for better health.

Drawing upon the SEM, this study broadened its scope to encompass a range of personal factors, thereby enabling a thorough examination of how various factors—including chronic diseases resources, fear of recurrence, hope, health locus of control—shape the self-management behaviors among post-stroke patients.

## 2 Materials and methods

### 2.1 Study design

This study adopts a cross-sectional study design, following the STROBE checklist for cross-sectional studies.

### 2.2 Participants

A total of 262 post-stroke patients were recruited from a tertiary hospital in Sichuan Province, based on the following inclusion criteria: aged ≥ 18 years; diagnosed with stroke for more than 2 weeks; in stable condition with a Barthel Index score greater than 40; conscious and able to communicate with researchers via text or voice; and provided informed consent. Exclusion criteria included participants with cognitive or mental disorders, or in extremely critical condition, such as multiple organ dysfunction.

The sample size was determined using Kendall’s rule (37), which recommends a sample size exceeding ten times the number of variables. This study includes 26 variables; therefore, the estimated minimum sample size was calculated to be 286 when accounting for a 10% efficiency adjustment.

### 2.3 Instruments

#### 2.3.1 Demographic and disease-related characteristics

The demographic and disease-related characteristics collected from participants include the following: age, gender, educational level, marital status, employment status, household per capita monthly income, method of medical expense payment, type of stroke, duration of stroke, ability to perform daily living activities (as measured by the Barthel Index), presence of comorbidities, oral medication regimen, and rehabilitation treatment status.

#### 2.3.2 Self-management behavior scale for post-stroke patients

SMBS-stroke was developed by Na et al. (38), including symptom management, daily life management, emotional management, utilization of resources, and rehabilitation exercise, with a total of 26 items. The scale uses a 5-point Likert scoring system, with scores ranging from 26 to 130. A higher score indicates an enhanced ability for patients to comprehend and execute self-management behaviors. The content validity of the scale is 0.857. The Cronbach’s α coefficients for the total scale and its five dimensions range from 0.708 to 0.982, and the split-half reliability ranges from 0.684 to 0.985 (38).

#### 2.3.3 Chronic illness resource scale

CIRS was developed by Glasgow et al. (21), and the Chinese version of the simplified CIRS was translated and revised by Li (39). This study uses the 22-item simplified CIRS revised by Xiaoyue (40), and adjusted for characteristics of stroke. The scale comprises 22 items across 7 dimensions, and uses a 5-point Likert scoring system, with scores from 1 to 5 representing “never” to “always.” Typically, a mean score of 3 is used as the threshold value to determine whether CIR is considered ideal; specifically, a score of 3 or above indicates an ideal status, while a score below 3 signifies suboptimal conditions. The Cronbach’s α coefficient for the CIRS in this study is 0.874 with each dimension coefficient ranging from 0.605 to 0.73.

#### 2.3.4 Fear of recurrence inventory short form

The Fear of Recurrence Inventory (FRI) was developed by Simard and Savard (41) in 2009 to access cancer patients’ fear of recurrence with a total of 42 items. The Chinese version of this inventory was revised by Ting (26). In 2015, Simard and Savard (27) identified that the FRI was not convenient for use due to its extensive number of items; therefore, they utilized the subscale of severity of the FRI as a shorter form of the FRI (FRI-SF) for rapid screening of fear of recurrence. The FRI-SF uses a 5-point Likert scoring system with a total of 9 items, where item 5 is scored reversely; a higher total score indicates a more severe fear of recurrence among patients.

#### 2.3.5 Herth hope index

HHI was developed by Herth (42) in 1992, which comprises a total of 12 items and employs a 4-point Likert scoring system, where a score of 1 represents strong disagreement and a score of 4 signifies strong agreement; it is important to note that items 3 and 6 are scored in reverse. A higher total score reflects an greater level of hope. The content validity index for the Chinese version is reported at 0.97 (43).

#### 2.3.6 Multidimensional health locus of control

MHLC was developed by Wallston et al. (33) in 1978 and translated by Ip and Martin (44) scale was recognized as the most widely used assessment tool for health psychological control sources and encompasses three dimensions, comprising a total of 18 items: Internal Health Locus of Control (ILC) with 6 items, Chance Health Locus of Control (CHLC) with 6 items, and Powerful Others Health Locus of Control (PHLC) with 6 items. The MHLC employs a 6-point Likert scale, allowing for separate calculation of total scores for each dimension; the highest score indicates the client’s predominant health locus of control type. The Cronbach’s α coefficients for the three dimensions range from 0.75 to 0.83, while test-retest reliability values fall between 0.66 and 0.71 (44).

### 2.4 Statistical analysis

Frequency distribution was used to characterize the categorical variables related to demographic and disease-related characteristics. Following assessments of skewness and kurtosis, Age, the scores for SMBS-stroke, a part of dimension of CIRS (including Physician/health care team, Family and friends, Personal, Neighborhood/community, Media and policy, Work), Hope, Fear of recurrence, Internal locus of control, and Powerful others health locus of control were found to be approximately normally distributed and described by the mean and standard deviation (SD). As the Organizations resource in CIRS and Chance health locus of control deviated from the normal distribution, we described them using the median and quartiles.

Based on the outcomes of normality tests and variance homogeneity tests, *t*-tests, ANOVA (using the LSD method), and Kruskal-Wallis H tests were conducted for single-factor analysis. The Pearson correlation analysis is used for the correlation analysis between variables that conform to the normal distribution; otherwise, the Spearman correlation analysis is used. Multiple linear regression analysis was applied to identify factors influencing self-management behaviors. All *P*-values were computed as two-tailed with a significance level at 0.05. Statistical analyses were carried out using SPSS version 26.0.

### 2.5 Ethics

The study has been approved by the biomedical ethics committee of the relevant hospital [No. 2020(1031)], and followed the principles outlined in the 1964 Helsinki Declaration and its later amendments. Informed consent was obtained from all participants. Prior to signing the informed consent form, participants were provided with a detailed explanation of the study’s purpose and procedures. They were informed that participation was entirely voluntary and that they could withdraw at any point during the survey. All collected data are anonymized to ensure that no information could identify individual participants; these data will remain confidential and will be utilized solely for research purposes.

## 3 Results

### 3.1 Demographic and disease-related characteristics

In this study, 300 questionnaires were distributed on-site at the outpatient departments of neurology or rehabilitation wards, resulting in a response rate of 296 (98.7%). And 262 responses were deemed valid, yielding an efficiency rate of 88.5%.

The demographic characteristics of the participants revealed that the majority were male (80.5%), with the average age of (53.13 ± 11.73). The educational attainment was predominantly at the junior high school/technical secondary school level (53.8%), while those holding a college degree or higher constituted 32.7%. Additionally, a significant proportion (90.5%) were married; however, most patients were not employed (72.9%). Furthermore, nearly half (49.2%) reported a household per capita monthly income exceeding 3,000 yuan, and an overwhelming majority (87.8%) had medical insurance.

Among the 262 patients included in this study, ischemic post-stroke individuals represented 71.8%, with those experiencing disease duration of less than 3 months accounting for 48.1%. Notably, 62.2% exhibited a Barthel Index score above 60, indicating mild dependence or independence in daily living activities; additionally, 84.4% had underlying conditions such as hypertension, diabetes mellitus, and hyperlipidemia; furthermore, 85.4% reported taking three or more oral medications; and finally 62.7% had undergone rehabilitation treatment. For further details regarding general information about the participants, please refer to Table 1.

**TABLE 1:** Demographic and disease-related characteristics of stroke patients (*n* = 262).

Categories	*N* = 262	%	Self-management behavior			
			**Mean**	**SD**	***F/t/Z***	***P***
**Gender^b^**						
Male	211	80.5	92.31	14.45	0.178	0.859
Female	51	19.5	91.90	15.36		
**Education level^a^**						
Primary school or below	35	13.4	82.63	14.79	17.416	<0.001
Secondary/High school	141	53.8	90.93	12.89		
Junior college or above	86	32.8	98.27	14.71		
**Marital status^b^**						
Married	237	90.5	92.02	14.41	–0.709	0.479
Unmarried/divorced/widowed	25	9.5	94.20	16.55		
**Employment status^b^**						
Employed	71	27.1	89.58	15.42	1.799	0.073
Unemployed/retired	191	72.9	93.21	14.20		
**Household per capita monthly income (Yuan)^a^**						
< 2,000	54	20.6	85.26	13.61	8.180	<0.001
2,000∼3,000	79	30.2	94.20	12.41		
> 3,000	129	49.2	93.94	15.45		
**Method of medical expense payment^b^**						
Medical insurance	230	87.8	92.97	14.46	–2.230	0.027
Self-paying	32	12.2	86.88	14.74		
**Type of stroke^b^**						
Hemorrhagic stroke	74	28.2	95.93	15.44	–2.603	0.010
Ischemic stroke	188	71.8	90.77	14.04		
**Duration of stroke (month)^a^**						
<3	126	48.1	93.37	14.70	0.821	0.441
3∼6	63	24.0	91.70	13.37		
> 6	73	27.9	90.71	15.47		
**Barthel index^c^**						
Moderate dependent	99	37.8	94.59	13.58	21.977	<0.001
Slight dependent	109	41.6	93.96	15.53		
Independent	54	20.6	84.41	11.80		
**Comorbidity^b^**						
Yes	221	84.4	91.63	14.46	1.548	0.123
No	41	15.6	95.46	15.12		
**Oral medication regimen^a^**						
< 3	38	14.5	93.50	16.48	0.769	0.464
3∼5	172	65.6	91.42	14.08		
> 5	52	19.8	93.96	14.93		
**Rehabilitation treatment status^b^**						
Yes	175	66.8	96.23	14.53	–6.811	<0.001
No	87	33.2	84.1	11.06		

^a^ANOVA.

^b^*t*-test.

^c^Kruskal-Wallis H test.

### 3.2 SMBS-stroke, CIRS, FRI-SF, HHI, MHLC of post-stroke patients

As demonstrated in Table 2, in this study, the total score for the SMBS-stroke ranged from 60 to 115, with a mean total score of 92.23 ± 14.61 and an item mean score of 3.55 ± 0.56. The five dimensions were ranked according to their item mean scores as follows: symptom management (3.73 ± 0.55), daily life management (3.61 ± 0.65), rehabilitation exercise (3.52 ± 1.09), emotional management (3.44 ± 0.61), and utilization of resources (3.08 ± 0.75).

**TABLE 2:** Self-management behavior, chronic illness resources, fear of recurrence, hope and health locus of control for stroke patients (*n* = 262).

Categories	Mean	SD
Self-management behavior	3.55	0.56
Symptom management	3.73	0.55
Daily life management	3.61	0.65
Rehabilitation exercise	3.52	1.09
Emotional management	3.44	0.61
Utilization of resources	3.08	0.75
Chronic illness resources	3.01	0.55
Personal	3.67	0.80
Family and friends	3.50	0.85
Physician/health care team	3.23	0.82
Media and policy	3.13	0.74
Neighborhood/community	2.83	0.77
Work (*N* = 71)	3.03	0.90
Fear of recurrence	19.17	7.13
Hope	39.32	5.26
Health locus of control	–	–
Internal health locus of control	27.29	4.21
Powerful others health locus of control	27.74	4.02
**Categories**	**Median**	**(P_25_, P_75_)**
Chronic illness resources	–	–
Organizations	1.67	(1.00, 2.33)
Health locus of control	–	–
Chance health locus of control	14.00	(11, 22)

The item mean score for the total CIRS among post-stroke patients was 3.01 ± 0.55, with dimension rankings as follows: personal resources (3.67 ± 0.80), family and friends resources (3.50 ± 0.85), physician/health care team resources (3.23 ± 0.82), media and policy resources (3.13 ± 0.74), and neighborhood/community resources (2.83 ± 0.77). The organizational resources dimension scored a median value of 1.67 [1.00, 2.33]. Over 70% of post-stroke patients in this study are currently not employed; consequently, the dimension of work resources had a valid response rate of only 27.1%, with means of 3.03 (SD = 0.90), leading to its exclusion from ranking and subsequent analyses.

The average score for fear of recurrence among post-stroke patients in this study was 19.17 ± 7.13.

Additionally, the mean of HHI in post-stroke patients in this study was 39.32 ± 5.26.

In terms of Internal Health Locus Control scores among post-stroke patients in this study, the scores for ILC in post-stroke patients was 27.29 ± 4.21, PHLC was 27.74 ± 4.02, and CHLC were 14.00 (11, 22).

### 3.3 The influencing factors of self-management behaviors of post-stroke patients

Univariate analysis results are reported in Table 1, which showed that educational level (*F* = 17.416, *P* < 0.001), household per capita monthly income (*F* = 8.18, *P* < 0.001), method of medical expense payment (*t* = 2.23, *P* = 0.027), type of stroke (*t* = 2.603, *P* = 0.01), Barthel Index (*Z* = 21.977, *P* < 0.001), and rehabilitation training (*t* = 6.811, *P* < 0.001) were significantly related to SMBS-stroke. Pearson correlation analysis was employed to investigate the relationship between age and self-management behavior; however, the analysis did not yield a significant correlation (*r* = 0.095, *P* = 0.123).

Pearson correlation analysis results were reported in Table 3, which revealed a positive correlation between the total score of SMBS-stroke and the total score of CIRS (*r* = 0.481, *P* < 0.01) as well as all its dimensions; HHI (*r* = 0.221, *P* < 0.01), ILC (*r* = 0.328, *P* < 0.01), and PHLC (*r* = 0.252, *P* < 0.01). Conversely, there was a negative correlation with CHLC (*r* = –0.302, *P* < 0.01). The results are shown in Table 3.

**TABLE 3:** The correlations between self-management behavior and chronic illness resources, fear of recurrence, hope, health locus of control in stroke patients (*n* = 262).

Variable	Symptom management	Daily life management	Emotion management	Utilization of resources	Rehabilitation exercise	The total score of Self-management Behavior Scale
Total chronic illness resources^b^	0.538**	0.378**	0.471**	0.438**	0.184*	0.481**
Physician/health care team^a^	0.452**	0.299**	0.359**	0.347**	0.157**	0.390**
Family and friends^a^	0.385**	0.330**	0.330**	0.307**	0.238**	0.392**
Personal^a^	0.494**	0.356**	0.408**	0.398**	0.177**	0.442**
Neighborhood/community^a^	0.286**	0.192**	0.279**	0.228**	0.039	0.240**
Media and policy^a^	0.441**	0.260**	0.395**	0.340**	0.080	0.356**
Organizations^a^	0.288**	0.196**	0.293**	0.257**	0.130*	0.259**
Work (*N* = 71)^a^	0.384**	0.240*	0.313**	0.213	0.030	0.274**
Fear of recurrence^a^	–0.173**	–0.084	–0.415**	–0.119	0.213**	–0.103
Hope^a^	0.331**	0.128*	0.432**	0.198*	–0.072	0.221**
Health locus of control						
Internal locus of control^a^	0.341**	0.222**	0.394**	0.343**	0.126*	0.328**
Powerful others health locus of control^a^	0.262**	0.185**	0.238**	0.280**	0.108	0.252**
Chance health locus of control^b^	–0.358**	–0.248**	–0.344**	–0.271**	–0.093	–0.302**

*P < 0.05,

**P < 0.01,

***P < 0.001.

^a^Pearson correlation analysis.

^b^Spearman correlation analysis.

To ensure that potentially significant factors were not overlooked, variables theoretically recommended or demonstrating a univariate relationship with SMBS-stroke (*P* < 0.1) were included in the multiple linear regression analysis: educational level, employment status, household per capita monthly income, method of medical expense payment, type of stroke, Barthel Index, Rehabilitation treatment status, all dimensions of CIR except work resources, fear of recurrence, hope, and all dimensions of HLC.

The multiple regression analysis results (Table 4) indicate that approximately 56.2% of the variation in SMBS-stroke scores could be explained by the following factors: educational level (β = 0.222, *P* < 0.001), rehabilitation training (β = 0.444, *P* < 0.001), physician/health care team resources (β = 0.139, *P* = 0.007), family and friends resources (β = 0.160, *P* = 0.003), personal resources (β = 0.182, *P* = 0.002), fear of recurrence (β = –0.145, *P* = 0.001), ILC (β = 0.181, *P* < 0.001), and CHLC (β = –0.141, *P* = 0.004).

**TABLE 4:** Multiple linear regression of self-management behavior in stroke patients (*n* = 262).

Variable	*B*	SD	β	95%CI (lower, upper)	*t*	*P*
Education level	4.969	0.965	0.222	(3.069, 6.870)	5.149	<0.001
Rehabilitation treatment status	13.752	1.380	0.444	(11.035,16.470)	9.965	<0.001
Physician/health care team	0.825	0.301	0.139	(0.232, 1.417)	2.742	0.007
Family and friends	0.915	0.306	0.160	(0.312, 1.518)	2.987	0.003
Personal	1.116	0.364	0.182	(0.400, 1.832)	3.070	0.002
Fear of recurrence	–0.297	0.091	–0.145	(–0.476, –0.119)	–3.283	0.001
Internal locus of control	0.630	0.168	0.181	(0.298, 0.961)	3.745	<0.001
Chance health locus of control	–0.300	0.105	–0.141	(–0.506, –0.094)	–2.869	0.004

*R*^2^ = 0.562.

## 4 Discussion

The primary findings of this study indicate that post-stroke patients exhibit moderate levels of self-management behaviors. These scores are predominantly influenced by educational level, rehabilitation treatment status, comorbidity, CIRS (resources from physician/health care team, family and friends, as well as personal resources), fear of recurrence, and health locus of control (ILC and PHLC).

The self-management status of patients in this study was close to the score range of 65-73% observed in the studies by Yan (45), and Lingli (15), but was slightly better than the results reported by Lu (19) and Lian (46). At the level of dimension comparison, symptom management ranked highest, which was consistent with the findings of Lu (19) and Lian (46); while emotion management and resource utilization occupied the two lowest positions, confirming the research findings of Lian (46), but differing from those of Rongfang (20). Differences among studies might be attributed to various factors, including the time and location of data collection, as well as the demographic characteristics of the participants. For instance, in the study conducted by Lian (46), the proportion of participants with lower household income was relatively high, and approximately 50% of the patients were completely dependent in terms of Barthel index, which could explain the relatively lower self-management ability observed in that study. In the study by Lu (19), a higher proportion of participants were employed, the participants were investing more time and energy into their work, it might have affected their self-management behaviors. Generally, the self-management behavior of Chinese post-stroke patients is at a moderate to low level. Although post-stroke patients demonstrated the ability to effectively manage stroke-related symptoms, their ability to cope with emotional challenges and psychological pressure related to the condition remained inadequate. Additionally, these patients have not yet fully utilized available resources to address issues related to disease management.

The educational level and rehabilitation treatment status emerged as significant predictors of patient self-management behaviors, echoing findings from prior research studies (17, 20). A plausible explanation for this phenomenon was that post-stroke patients with higher education levels possess a greater capacity to accept and assimilate new knowledge and methods. This underscores the importance of healthcare professionals considering the individual circumstances of patients carefully and taking into account each patient’s ability to comprehend and process information while implementing personalized nursing strategies and humanized health education approaches. On the other hand, the systematic review conducted by Parke et al. (47) highlights that post-stroke rehabilitation treatments and patient self-management programs share several commonalities, including the enhancement of self-efficacy in problem-solving, the formulation of action plans, and decision-making processes. It suggests that given the current context in which post-stroke self-management programs are not widely adopted, professional rehabilitation treatment could offer more effective support for self-management to both post-stroke patients and their families.

This study also confirmed the positive impact of chronic illness resources on self-management behaviors among stroke patients, which is consistent with the conclusions of previous research (48). Furthermore, this study provides a detailed analysis of the influence of six dimensions of chronic illness resources. Multiple analysis reveals that the self-management behaviors of post-stroke patients were influenced by the resources from physician/health care team, family and friends and, most importantly, personal self. As the topmost factor in the SEM (21), personal resources are the determining factor on self-management behaviors, which mean external resources must be internalized into patients’ motivations to effectively influence their health-related behaviors. These findings suggested that, on one hand, supports from health care staff contributed to the early recovery of post-stroke patients, particularly through professional rehabilitation guidance (49). On the other hand, within the cultural context of Chinese families, family members emerge as one of the most significant sources of social support for patients. They often assume the role of caring for post-stroke patients, making profound contributions to recovery and disease management through activities such as assisting with daily living tasks, overseeing rehabilitation exercises, and alleviating emotional stress. However, ultimately, both healthcare providers and family caregivers should strive to minimize patients’ passive receipt of information in clinical settings, especially in situations involving health education and decision-making. Instead, they should urge patients to actively engage in their own health management and decision-making, fully utilizing each patient’s unique strengths.

Another interesting finding of this study was that, overall, the fear of experiencing recurrent stroke among post-stroke patients impedes their self-management. This observation contrasts with previous studies suggesting that a low level of fear regarding recurrence could be considered a normal emotional response to the consequences of the disease and might promote healthy behaviors (50). A possible explanation for this phenomenon was that the fear of recurrence in post-stroke patients was influenced by both the progression of the disease and their daily living activity capabilities (51), and could serve as an intermediary factor affecting patients’ self-management behaviors through other variables, which warrants further investigation in future research. Generally, it was evident that heightened fear of recurrence among post-stroke patients contributes to an increased perception of burden and diminishes their self-efficacy and quality of life—factors detrimental to effective disease self-management. Therefore, healthcare professionals should prioritize assisting post-stroke patients in developing a constructive perception of recurrence risk. Emphasis should be placed on educating them about knowledge and preventive measures related to recurrence risk while simultaneously working to alleviate their fears surrounding potential recurrences.

This study revealed that among healthy locus of control, the ILC positively predicts self-management behavior in post-stroke patients. While prior research has touched on this subject, Zirk and Storm (52) have concluded that ILC negatively affect depression, which partially align with the findings in this study. As a significant and stable personality variable, ILC encourages patients to actively seek social support and adopt constructive coping mechanisms, particularly in the face of adversity; this enables them to approach challenges with a more optimistic outlook (53). Conversely, post-stroke patients exhibiting higher scores on the Chance Health Locus of Control (CHLC) tend to be more fatalistic and often struggle to address problems independently. Therefore, it was imperative for healthcare professionals to promptly assess patients’ perceptions regarding disease management. By doing so, they could effectively motivate patients to harness their intrinsic drive for self-management, thereby enhancing their self-management behaviors.

Despite the significant findings, this study has certain limitations: it was a single-center survey that relied on patients’ self-reported data, which might introduce selection biases and reduce the representativeness of the sample; furthermore, the factors influencing post-stroke patients’ self-management behaviors are highly complex. This study only included a limited number of variables for quantitative analysis, and the explanations for the influencing paths and mechanisms were limited.

## 5 Conclusion

The self-management behaviors of post-stroke patients during the recovery period are generally observed to be at a moderate level, with emotional management and resource utilization identified as areas of weakness in their self-management practices. In terms of influencing factors, personal resources, the establishment of appropriate psychological control mechanisms, and the development of a positive perception regarding recurrence risk significantly contribute to effective self-management. Furthermore, patients’ acceptance of professional rehabilitation treatments and enhanced support from CIRS, including assistance from medical staff as well as family and friends, play crucial roles in this process.

## Data Availability

The raw data supporting the conclusions of this article will be made available by the authors, without undue reservation.
